# Epigenetic modifications inhibit the expression of MARVELD1 and in turn tumorigenesis by regulating the Wnt/β-catenin pathway in pan-cancer

**DOI:** 10.7150/jca.63608

**Published:** 2022-01-01

**Authors:** Jingchun Zhang, Qingwei Li, Qinliang Sun, Bojun Wang, Ying Cui, Changjie Lou, Yuanfei Yao, Yanqiao Zhang

**Affiliations:** 1 Department of Gastrointestinal Medical Oncology, Harbin Medical University Cancer Hospital, Harbin, Heilongjiang Province, China.; 2 Translational Medicine Research and Cooperation Center of Northern China, Heilongjiang Academy of Medical Sciences, Harbin, Heilongjiang Province, China.; 3 Department of Ultrasound, Second Affiliated Hospital of Harbin Medical University, Harbin, Heilongjiang Province, China.

**Keywords:** MARVELD1, prognosis, epigenetic modification, pan-cancer, Wnt pathway

## Abstract

MARVEL domain-containing 1 (MARVELD1) is one of the MARVEL domain-containing proteins. Expression of MARVELD1 in tumor and non-tumor tissues, the relationship between its expression and cancer prognosis, and upstream regulation of MARVELD1 were examined using pan-cancer data from The Cancer Genome Atlas. MARVELD1 expression was significantly downregulated in tissues used for pan-cancer analysis compared to that in normal tissues. Low expression of MARVELD1 was associated with poor disease outcomes in pan-cancer. Colon cancer patients with low expression of MARVELD1 had worse progression free survival and overall survival than those with high expression levels in our cohort. Hypermethylation and histone modification in the MARVELD1 promoter locus synergistically affected its expression in pan-cancer. The function of MARVELD1 in colon cancer remains to be studied. Gene Ontology enrichment analysis revealed that MARVELD1 may modulate processes associated with inhibition of tumorigenesis in colon cancer. Both upstream transcription factors and downstream functional enrichment of MARVELD1 were related to the Wnt/β-catenin signaling pathway. Overexpression of MARVELD1 inhibited the expression of β-catenin and its entry into the nucleus. MARVELD1 also inhibited the proliferation, migration, and invasion of colon cancer cells. With Wnt/β-catenin activator LiCl treatment, rescue experiments demonstrated that the role of MARVELD1 in colon cancer progression was dependent on the Wnt/β-catenin pathway. These results indicate that MARVELD1 acts as a tumor suppressor and inhibits tumorigenesis via the Wnt/β-catenin pathway.

## Introduction

Epigenetics, a branch of genetics, is defined as the study of heritable alterations in gene expression that do not cause permanent changes in the DNA sequence, including DNA methylation, histone modification, chromosome remodeling, and non-coding RNA regulation 1. Genetic and epigenetic aberrations, leading to the activation of oncogenes and inactivation of tumor suppressor genes, have been considered to play major roles in the pathogenesis of various cancers 2, 3.

MARVEL domain-containing 1 (MARVELD1) is a member of the MARVEL (myelin and lymphocyte protein (MAL) and related proteins for vesicle trafficking and membrane link) domain-containing protein family. MARVELD1 is located on human chromosome 10q24 (a locus associated with multiple cancers), and it is downregulated in multiple primary tumors derived from the ovary, vulva, uterus, cervix, breast, testis, kidney, bladder, and liver 4, 5. MARVEL domain-containing proteins have attracted increasing interest because they exhibit tumor suppressor activities and are commonly decreased via promoter methylation in breast, cervical, prostate, hepatocellular, esophageal, and gastric carcinomas or cell lines 6-9. MARVELD1 inhibits cell proliferation, tumor growth, and chemosensitivity of HCC cells by increasing p53 and p16 both *in vitro* and *in vivo* 10. In lung cancers, the expression of MARVELD1 significantly correlated with diagnostic histopathology and degree of malignancy as it was inactivated via DNA hypermethylation and histone deacetylation synergistically but suppressed the epithelial-mesenchymal transition 10-11. However, the function of MARVELD1 in colon cancer is unknown. Further studies are required to elucidate the upstream regulation and biological functions of MARVELD1 in tumor progression.

This study was conducted to evaluate the 1) expression of MARVELD1 in human cancer tissues and adjacent normal tissues, 2) relationship between its expression and disease prognosis, and 3) upstream regulation of MARVELD1 using pan-cancer data from The Cancer Genome Atlas (TCGA). In our cohort of patients with colon cancer, low expression of MARVELD1 indicated poor prognosis. Therefore, we aimed to elucidate the downstream regulatory functions of MARVELD1 and the correlation among MARVELD1 and key genes of important pathways in colon cancer. Collectively, our findings indicate that DNA methylation and histone modification synergistically affect the expression of MARVELD1, which inhibited tumor occurrence and progression via Wnt/β-catenin signaling.

## Materials and Methods

### Acquisition of Public Date

Genome-wide expression profiles of MARVELD1 as well as other genes and the clinicopathological progression were downloaded from TCGA (https://tcga-data.nci.nih.gov/). MARVELD1 expression levels were dichotomized using the median expression as the cutoff to define “high value” at or above the median versus “low value” below the median. The log-rank test was used to examine the survival difference between different patient groups.

The DNA methylation profile was measured experimentally using the Illumina Infinium HumanMethylation450 (450K) platform. It was downloaded from TCGA database. Beta values were obtained from Johns Hopkins University and University of Southern California TCGA genome characterization center. DNA methylation values, described as beta values, were recorded for each array probe in each sample via BeadStudio software (Illumina). DNA-methylation beta values were continuous variables between 0 and 1, representing the ratio of the intensity of the methylated bead type to the combined locus intensity. Thus, higher beta values represent higher levels of DNA methylation and lower beta values represent lower levels of DNA methylation.

### Transcription Factor Prediction

Transcriptional factors were predicted using JASPAR software (jaspar.binf.ku.dk). Geneontology term enrichment (GO) and Kyoto Encyclopedia of Genes and Genomes (KEGG) pathway analysis were analysed for these TFs using DAVID Functional Annotation Bioinformatics Microarray Analysis (https://david.ncifcrf.gov/) (12, 13). ChIP-seq data were downloaded from the ENCODE database (https://www.encodeproject.org/).

### Functional Enrichment Analysis

Pairwise Pearson correlation analysis was performed to determine the expression of MARVELD1 and all other genes. Only positively correlated genes with an R ≥ 0.6 and a significant correlation (P < 0.05) were retained. Gene Ontology (GO) and Kyoto Encyclopedia of Genes and Genomes (KEGG) pathway analyses were performed using DAVID Functional Annotation Bioinformatics Microarray Analysis (https://david.ncifcrf.gov/).

### Ethics approval, Patient consent, and Tissue samples

Colon cancer tissues and the corresponding adjacent normal colon tissues, for qRT-PCR, were collected from patients at the Third Affiliated Hospital of Harbin Medical University. The specimens were frozen in liquid nitrogen and stored at -80 °C until use.

Tissue samples from advanced or metastatic colon cancer patients, diagnosed between January 2008 and December 2012, and from pancreatic cancer patients, diagnosed between January 2010 and December 2015, were obtained for immunohistochemistry at the Third Affiliated Hospital of Harbin Medical University. These samples met the following requirements: (1) the paraffin-embedded pathological tissue specimens were well preserved, and (2) the basic information and clinicopathological data were complete. (3) All patients have complete follow-up records. (4) All patients received chemotherapy after surgery. The chemotherapy regimen for pancreatic cancer is based on gemcitabine. The chemotherapy regimen for colon cancer is fluorouracil and oxaliplatin (patients with stage I colon cancer do not need chemotherapy after surgery and were not included in this study).

The studies involving human participants were reviewed and approved the Research Ethics Committee of the Third Affiliated Hospital of Harbin Medical University. All patients provided written informed consent, and the specimens were handled in accordance with accepted ethical standards.

### Immunohistochemistry

The samples were cut into sections (5 μm), and the sections were de-paraffinized in xylene, rehydrated in ethanol, and washed in phosphate-buffered saline (PBS). The sections were incubated with anti‐MARVELD1 antibody (TA351385, OriGene) overnight at 4 °C followed by incubation with a secondary antibody (Beijing Zhongshan Jinqiao Biotechnology Co., Ltd.) for 30 min at room temperature. The samples were observed and photographed using a light microscope (Olympus). Three pathology professionals scored the expression of MARVELD1 in the tissues was scored by immunoreactive score (IRS), and 8 points were selected as the cut-off value, and they were divided into high expression group and low expression group. According to the patient's follow-up information, the correlation between MARVELD expression and the patient's prognosis was analyzed. All patients provided written informed consent, agreed to use their organization for this study, and the specimens were handled in accordance with accepted ethical standards.

### Cell Culture

All cell lines were obtained from the Chinese Type Culture Collection, Chinese Academy of Sciences. The cells were cultured in RPMI-1640 (Gibco, Rockville) or DMEM (Gibco, Rockville) medium, that were supplemented with 10% fetal bovine serum (FBS) and 100 U/ml of penicillin/streptomycin at 37 °C in an atmosphere containing 5% CO_2_. Cells in the logarithmic growth phase were used. The Wnt/β-catenin activator LiCl was obtained from Sigma-Aldrich (Munich, Germany). HCT116 and HT29 cells with MARVELD1 overexpression or control transfection were cultured in medium with or without 20 mmol/L LiCl.

### Plasmid Transfection and Lentivirus Infection

MARVELD1 ORF region was cloned into the vector pcDNA3.1/flag (Invitrogen, Carlsbad). The cells were transfected with the MARVELD1 construct using Lipofectamine 2000 (11668-019, Invitrogen) according to the manufacturer's protocol. The negative control and MARVELD1 over-expressing lentiviruses/flags were purchased from Invitrogen. Next, lentiviruses infected 5 × 10^5^ cells in a 6-well plate with 4-6 µg/mL polybrene (107689, Sigma). After 24 h, the infected cells were subjected to selection with 4 µg/ml puromycin (540411, Calbiochem) and cultured for 3 days. Stable overexpression cell lines were identified using qRT-PCR and western blot assays.

### Transfection of Small Interfering RNAs

A scrambled negative control small interfering RNA (siRNA) and MARVELD1 siRNAs were purchased from Invitrogen. The cells were transfected with the siRNAs using Lipofectamine 2000 according to the manufacturer's protocol. The sequences were as follows: siMARVELD1#1: 5′-AUUGGAACCAGGCUUCUGGTT-3′; siMARVELD1#2: 5′-CCAGAAGCCUGGUUCCAAUTT-3′.

### Cell Viability Assays

The viability of treated cells was measured using a Cell Counting Kit-8 (CCK-8; CK04; Dojindo Laboratories, Kumamoto, Japan) according to the manufacturer's instructions. The cells (1 × 10^3^) were cultured at 37 °C in a 96-well plate. Cell viability was assessed at 24, 48, and 72 h. After 10 μL CCK-8 solution was added to each well, the plates were incubated at 37 °C for 1 h. A microplate reader was used to measure the absorbance of each cell suspension at 450 nm. RPMI-1640 medium containing 10% FBS and 10% CCK-8 was used as the control.

### Colony Formation Assays

Cells were plated in a 6-well plate with 1,000 cells/well and cultured in RPMI-1640 medium containing 10% FBS for 2 weeks. Cell colonies were washed with PBS, fixed with 4% paraformaldehyde, stained with 0.5% crystal violet (332488, Sigma-Aldrich) for 30 min. The cells counted under a microscope for quantitative analysis.

### Invasion Assays

The transwell chambers were first coated with Matrigel solution. HCT116 and HT29 cells lines in serum-free RPMI-1640 medium were placed in the BRAND® insert with Matrigel (BR782806, Sigma-Aldrich). RPMI-1640 medium containing 20% FBS was added to the lower chamber. After incubating at 37 °C for 24 h, the invading cells that had penetrated through the lower surface of the membrane were fixed with 4% paraformaldehyde for 30 min. The cells were stained with 0.5% crystal violet for 30 min and were counted in six randomly chosen fields under a light microscope.

### Wound Healing Assay

The cells were plated in 6-well culture plates and cultivated to achieve over 90% confluence in RPMI-1640 medium containing 10% FBS. A vertical wound per well was created using a 10 µL pipette tip. After 2 washes with PBS to eliminate cell debris, the cells were re-incubated in RPMI-1640 containing 5% FBS. Images were recorded at the indicated time to measure the size of the remaining wound.

### RNA Preparation and qRT-PCR

Total RNA was extracted using TRIzol Reagent (15596018, Invitrogen) according to the manufacturer's protocol. The Transcriptor First Strand cDNA Synthesis Kit (Roche, Mannheim, Germany) was used to reverse transcribe RNA into complementary DNA. mRNA expression was quantified via real-time PCR using FastStart Universal SYBR Green Master Mix (04913850001, Roche) with gene-specific primers, with GAPDH as the internal control. The results were determined using the comparative CT (2-ΔΔCT) method. The forward (F) and reverse (R) primer sequences were as follows: MARVELD1-F: 5′ -ACTGAGAAGTCCCGCTGTTACAG-3′; MARVELD1-R: 5′-GGGATGCTGGGAATCTTAAGG-3′; GAPDH-F: 5′-CATGTTCGTCATGGGTGTGAA-3′; GAPDH-R: 5′-GGCATGGACTGTGGTCATGAG-3′.

### Western Blot Analysis

Total cell lysates were harvested from cultured cells using ice-cold lysis buffer. Nuclear and Cytoplasmic Protein Extraction Kit (WLA020a, Wanleibio) was used to extract the nuclear and cytoplasmic proteins. Protein concentrations were evaluated using a protein assay kit (5000001, Bio-Rad), and equal amounts of protein were separated using 10% SDS-PAGE, followed by electroblotting onto PVDF membranes, which were blocked with 5% non-fat milk in PBS with 0.1% Tween-20 (Tween20-PBS) for 2 h at room temperature. The membranes were immunoblotted with primary antibodies against MARVELD1 (TA351385, origin), β-catenin (Proteintech), Lamin B1 (13435S; Cell Signaling Technology), flag tag (66008-3-lg, Proteintech), and GAPDH (TA-08, ZSGB-BIO) overnight at 4 °C. After washing with Tween20-PBS, the membranes were incubated with horseradish peroxidase (HRP)-conjugated secondary antibodies (Proteintech) for 1 h. Immunoreactive proteins were detected using a chemiluminescence solution (Thermo Fisher Scientific).

### Animal Experiments

The animal study was reviewed and approved by the Medical Experimental Animal Care Commission of Harbin Medical University. Written informed consent was obtained from the owners for the participation of their animals in this study. Four-week-old Balb/c mice were obtained from Hunan Slac Jingda Laboratory Animal Co, Ltd. Approximately 2 × 10^6^ stably overexpressing cells, i.e., HCT116-controls or HCT116-flagMARVELD1, in 200 μL of serum-free RPMI-1640 medium were injected directly into the right back of mice using an 1-mL syringes and 20-gauge needles. The tumor volume was calculated using the following formula: (length × width^2^)/2. The mice were euthanized, and the tumor weight was examined.

### Statistical Analysis

The expression and epigenetic modifications of MARVELD1 in cancer tissues compared to those in adjacent non-cancerous tissues were examined by a t-test. The Kaplan-Meier method and a log-rank test were used to evaluate the difference in survival between patients with high and low MARVELD1 expression. The differences among groups in the results of *in vitro* and *in vivo* experiments were also analyzed using Student's t-test. All statistical tests were two-tailed, and P < 0.05 indicated statistical significance. Statistical analyses were performed using R.3.5.3 software.

## Results

### MARVELD1 expression was downregulated and positively associated with prognosis of patients in pan-cancer database

**Figure 1:**
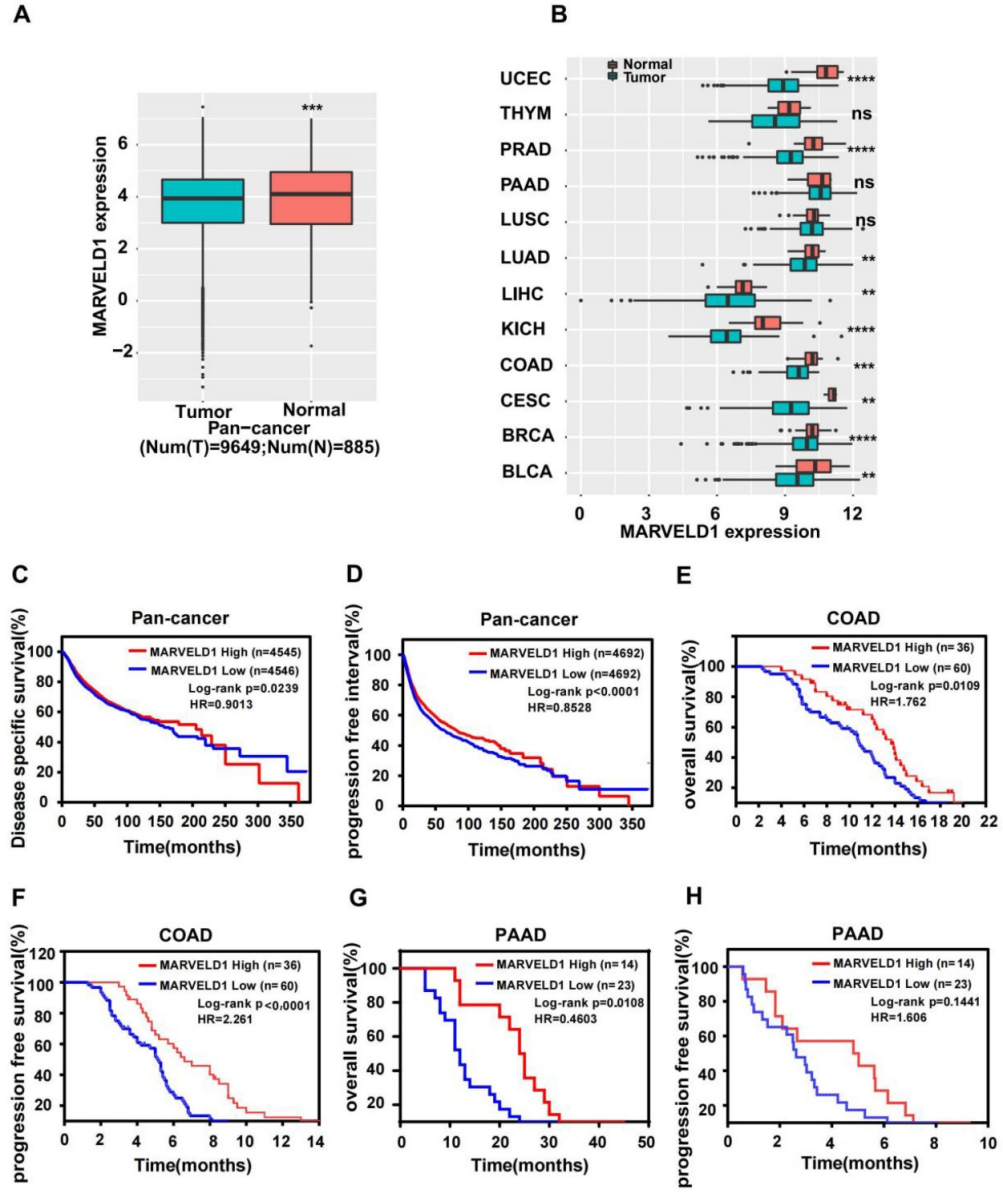
** Expression of MARVEL domain-containing 1 (MARVELD1) in tumor and non-tumor tissues, the relationship between its expression and cancer prognosis in human cancers. (A)** MARVELD1 expression is lower in the cancer cohort than in the corresponding normal tissues as observed in the data from The Cancer Genome Atlas (TCGA). **(B)** MARVELD1 expression is lower in UCEC, PRAD, LUAD, LIHC, KICH, COAD, CESC, BRCA, and BLCA than in the corresponding normal tissues. (C,D) Low expression of MARVELD1 correlates with poor disease specific survival (DSS) and progression free interval (PFI) according to TCGA pan-cancer database. **(E,F)** Low expression of MARVELD1 in COAD correlates with poor overall survival (OS) and progression free survival (PFS) in our cohort. **(G,H)** Low expression of MARVELD1 in PAAD correlates with poor OS in our cohort. *p< 0.05; **p < 0.01; ***p < 0.001; ****p < 0.0001; ns, no significance.

TCGA data were downloaded and analyzed to assess the expression of MARVELD1 in human cancers. Data from 10,534 patients representing 33 cancer types were obtained from TCGA pan-cancer database and were used to validate the expression of MARVELD1. The expression of MARVELD1 was significantly downregulated in cancer tissues compared to that in normal tissues in pan-cancer (Figure 1A; Supplementary Table 1). MARVELD1 expression was significantly lower in most types of cancers, including bladder urothelial carcinoma (BLCA), breast invasive carcinoma (BRCA), endocervical adenocarcinoma (CESC), colon adenocarcinoma (COAD), kidney Chromophobe (KICH), liver hepatocellular carcinoma (LIHC), lung adenocarcinoma (LUAD), prostate adenocarcinoma (PRAD), and uterine corpus endometrial carcinoma (UCEC), than in their respective non-cancer tissues (Figure 1B, Supplementary Table 1). This result was consistent with previous reports. Moreover, in our cohort, the expression of *MARVELD1* was lower in colon cancer samples than in their corresponding non-cancer counterparts (Supplementary Figure 1A,B). The results indicate that MARVELD1 was mainly located in the nucleus in colon cancer, which was consistent with previous reports 5. Kaplan-Meier analysis and the log-rank test were used to explore the relationship between MARVELD1 expression and survival status in patients using pan-cancer database samples. The results revealed that patients with low MARVELD1 expression had poor disease-specific survival (DSS) and progression-free interval (PFI) than those with high expression levels (Figure 1C,D). Colon cancer and pancreatic cancer were analyzed in our cohort. In the colon cancer group, there were 60 and 36 patients with low and high expression of MARVELD1, respectively (Supplementary Figure 1C). Downregulation of MARVELD1 in colon cancer was associated with both poor overall survival (OS) and progression free survival (PFS) unlike in cancers with high expression of MARVELD1 (Figure 1E,F, Supplementary Table 2). Low expression of MARVELD1 in pancreatic cancer was associated with poor OS unlike in cancers with high expression levels of MARVELD1 in our cohort (Figure 1G,H, Supplementary Table 3).

### Epigenetic modification of MARVELD1

**Figure 2:**
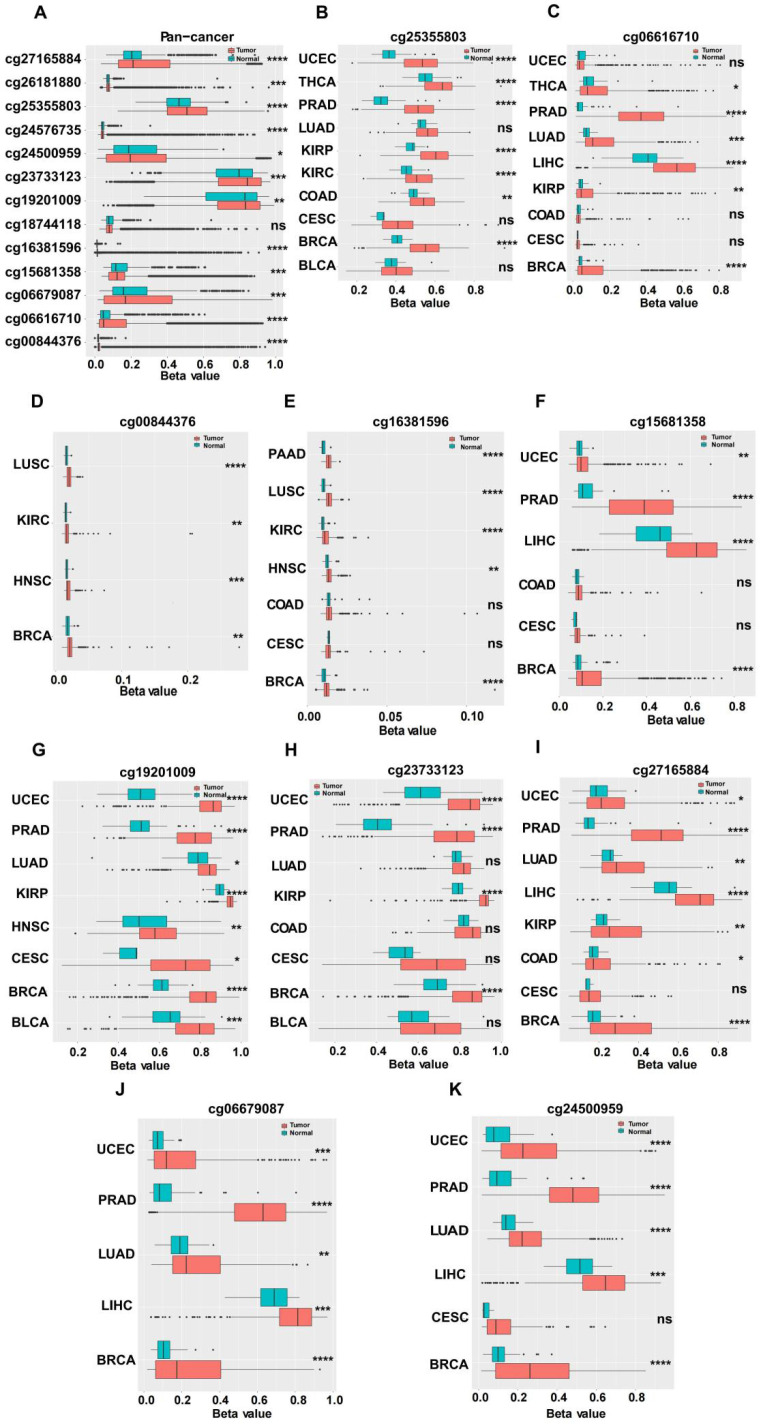
** Hypermethylation in promoter locus of MARVEL domain-containing 1 (MARVELD1) in human cancers compared to their non-cancer counterparts from The Cancer Genome Atlas (TCGA). (A)** Methylation is higher in the MARVELD1 promoter locus at 13 methylation sites in human cancers, including cg27165884, cg26181880, cg25355803, cg24576735, cg24500959, cg23733123, cg19201009, cg18744118, cg16381596, cg15681358, cg06679087, cg06616710, and cg00844376. **(B-K)** Every methylation site in the MARVELD1 promoter locus was significantly hypermethylated than in its non-cancer counterpart in most types of cancers. All statistical tests were two-tailed. *p< 0.05; **p < 0.01; ***p < 0.001; ****p < 0.0001; ns, no significance.

**Figure 3:**
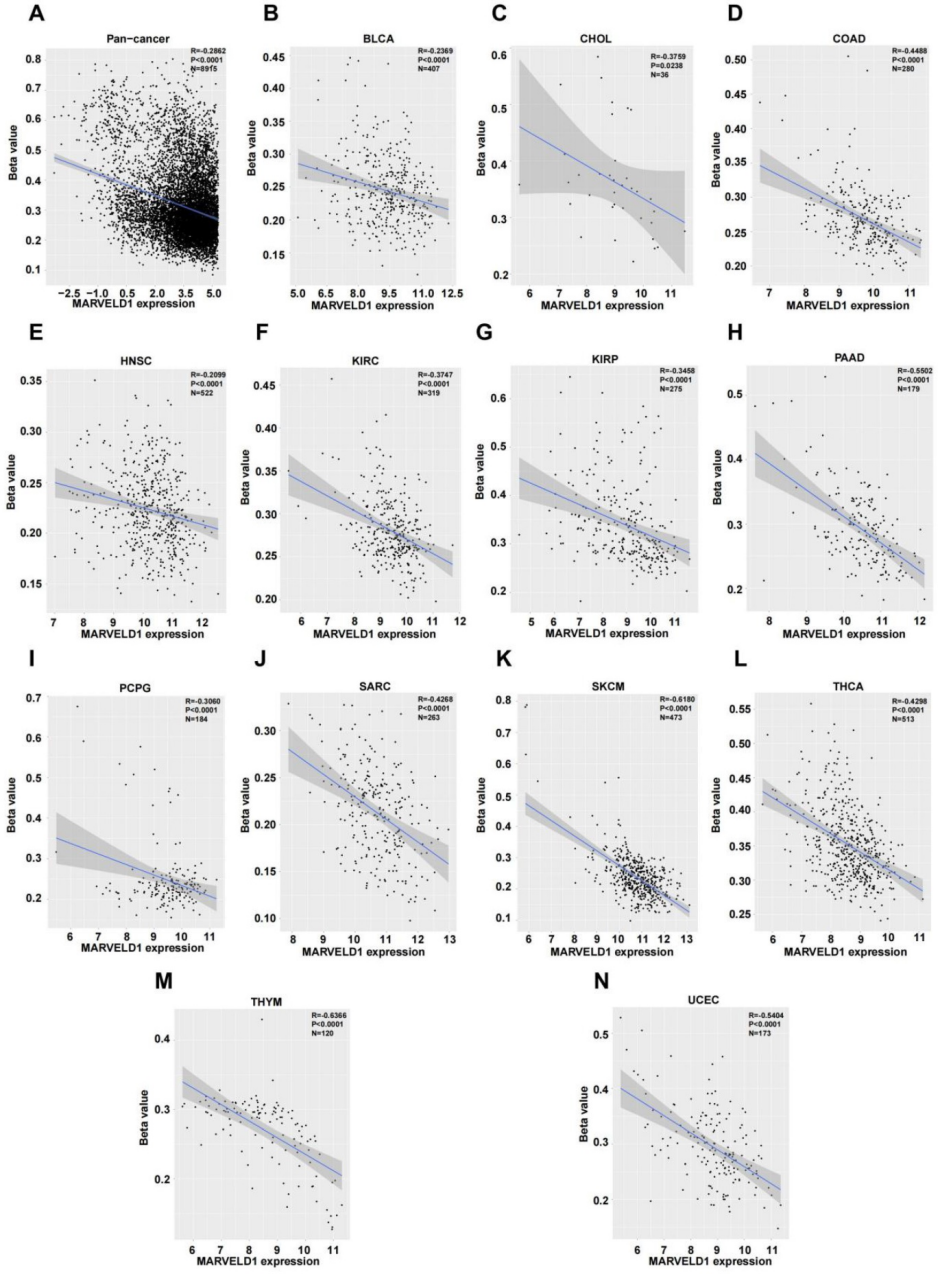
** MARVEL domain-containing 1 (MARVELD1) promoter locus hypermethylation is inversely correlated with MARVELD1 expression in human cancer. (A)** MARVELD1 promoter locus hypermethylation is inversely correlated with MARVELD1 expression in a pan‐cancer cohort. **(B-N)** MARVELD1 promoter locus hypermethylation is inversely and significantly correlated with MARVELD1 expression in BLCA, CHOL, COAD, HNSC, KIRC, KIRP, PAAD, PCPG, SARC, SKCM, THCA, THYM, and UCEC from The Cancer Genome Atlas (TCGA) database. All statistical tests were two-tailed, and P < 0.05 indicated statistical significance.

We investigated the upstream regulation of MARVELD1. As epigenetic regulation had been identified as a common type of control in gene expression 14. In this study, we used TCGA pan-cancer database to further analyze the correlation between the expression of MARVELD1 and epigenetic modifications. Data from the 450K platform and ENCODE were downloaded from TCGA portal to predict 13 methylation sites in the promoter locus of MARVELD1. These sites included cg27165884, cg26181880, cg25355803, cg24576735, cg24500959, cg23733123, cg19201009, cg18744118, cg16381596, cg15681358, cg06679087, cg06616710, and cg00844376. As expected, significant hypermethylation of the 13 MARVELD1 methylation sites was observed in the tissues from the pan-cancer data compared to the non-cancer tissues from TCGA (Figure 2A). Every methylation site in the MARVELD1 promoter locus was significantly hypermethylated than in its non-cancer counterpart in most types of cancers (Figure 2B-K). Data from 19,639 patients representing 33 cancer types obtained from TCGA were also examined to analyze the relationship between the methylation status of MARVELD1 promoter locus and its corresponding expression. The results indicated that MARVELD1 expression was inversely proportional to the DNA methylation level found in the pan-cancer data (Figure 3A). Moreover, MARVELD1 promoter locus hypermethylation inversely and significantly affected the expression of MARVELD1 in BLCA, cholangio carcinoma (CHOL), COAD, head and neck squamous cell carcinoma (HNSC), kidney renal clear cell carcinoma (KIRC), kidney renal papillary cell carcinoma (KIRP), pancreatic adenocarcinoma (PAAD), pheochromocytoma and paraganglioma (PCPG), sarcoma (SARC), skin cutaneous melanoma (SKCM), thyroid carcinoma (THCA), thymoma (THYM), and UCEC (Figure 3B-N). These specific data, including the number of patients and the P values, are shown in Tables 1 and 2.

**Figure 4:**
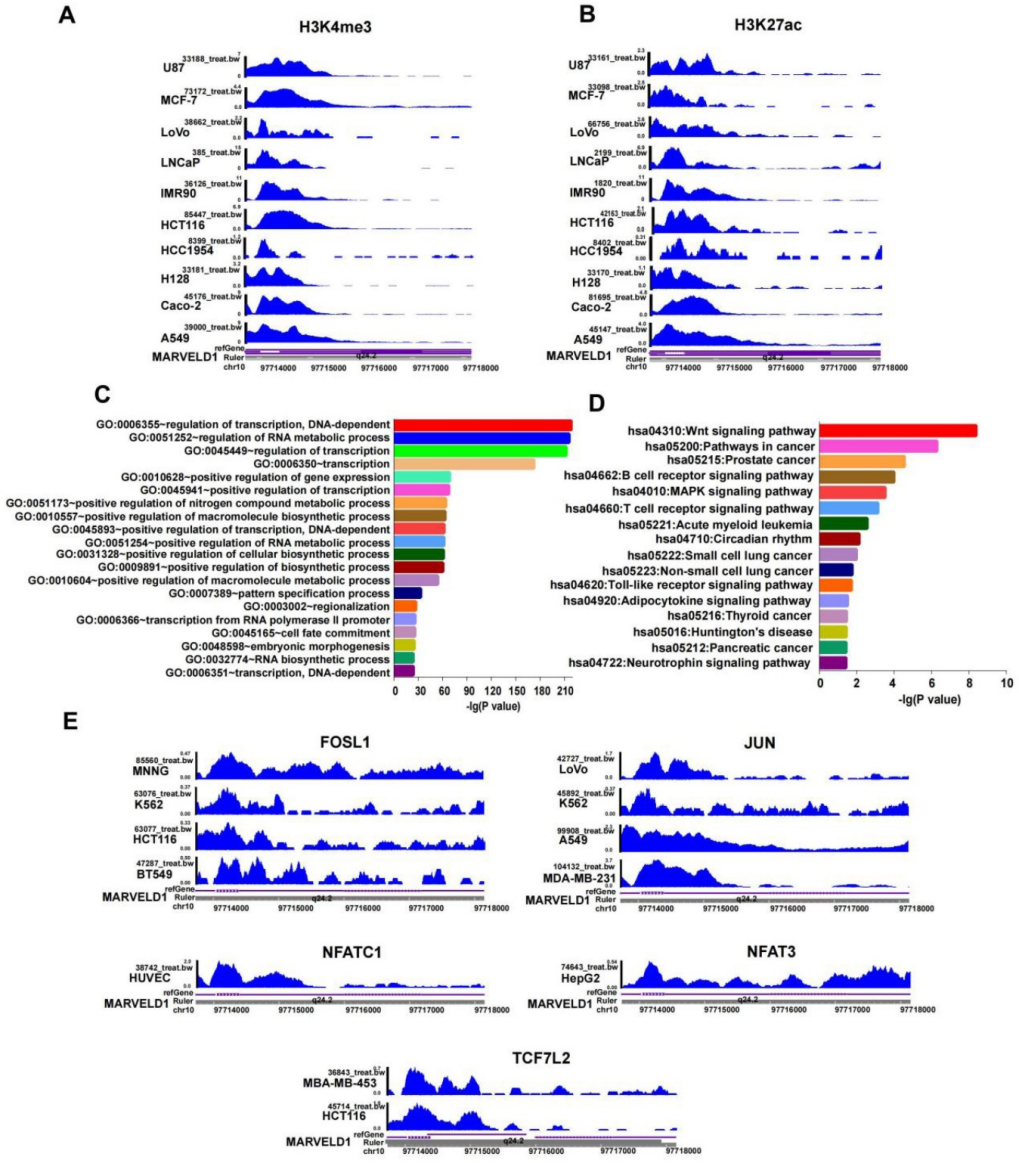
** Histone modification and transcription factor (TFs) bind to the MARVEL domain-containing 1 (MARVELD1) promoter locus are observed in the MARVELD1 promoter locus. (A-B)** H3K27ac and H3K4me3 enrichment in the MARVELD1 promoter locus are observed in U87, MCF-7, LoVo, LNCaP, IMR90, HCT116, HCC1954, H128, Caco-2, and A549 cell lines. **(C-D)** Gene Ontology (GO) and Kyoto Encyclopedia of Genes and Genomes (KEGG) are used in function and pathway enrichment analysis for upstream TF binding to the MARVELD1 promoter locus. **(E)** FOSL1, JUN, NFAT3, NFATC1, TCF7L2 bind to the promoter region of MARVELD1 in most types of cancers cell lines.

In addition to DNA methylation, other epigenetic modifications, including H3K27ac and H3K4me3, were also studied to examine their roles in regulating MARVELD1 expression. The results indicated that H3K27ac and H3K4me3 were enriched in the MARVELD1 promoter locus in ten cancer cell lines representing 10 cancer types, namely U87, MCF-7, LoVo, LNCaP, IMR90, HCT116, HCC1954, H128, Caco-2, and A549 (Figure 4A,B). These results indicated that the low expression of MARVELD1 could be attributed to a biphasic regulation of DNA methylation and histone modification. The lower levels of MARVELD1 expression were related to the comprehensive regulation of epigenetic factors, such as DNA methylation and histone acetylation.

### Regulation of upstream transcription factors of the MARVELD1 promoter locus

JASPAR and TRANSFAC software were used to predict transcription factor (TF) binding to the MARVELD1 promoter locus. DAVID Functional Annotation Bioinformatics Microarray Analysis analyzed GO and KEGG pathways. The results indicated that transcription factors bound to the promoter region of MARVELD1 were related to the Wnt pathway. We then analyzed the crucial genes of the Wnt pathway according to KEGG, and screened five transcription factors, including FOSL1, JUN, NFATC1, NFAT3, and TCF7L2 (Figure 4C,D). To verify this result, we downloaded ChIP-seq data for FOSL1, JUN, NFATC1, NFAT3, and TCF7L2 from the ENCODE database. The results indicated that FOSL1 binds to the promoter region of MARVELD1 in BT549, HCT116, K562, and MNNG cell lines; JUN in MDA-MB-231, A549, K562, and LoVo cell lines; NFATC1 in HUVEC cell lines; NFAT3 in HepG2 cell lines; and TCF7L2 in HCT116 and MBA-MB-453 cell lines (Figure 4E).

### Pivotal biological functions of MARVELD1

**Figure 5:**
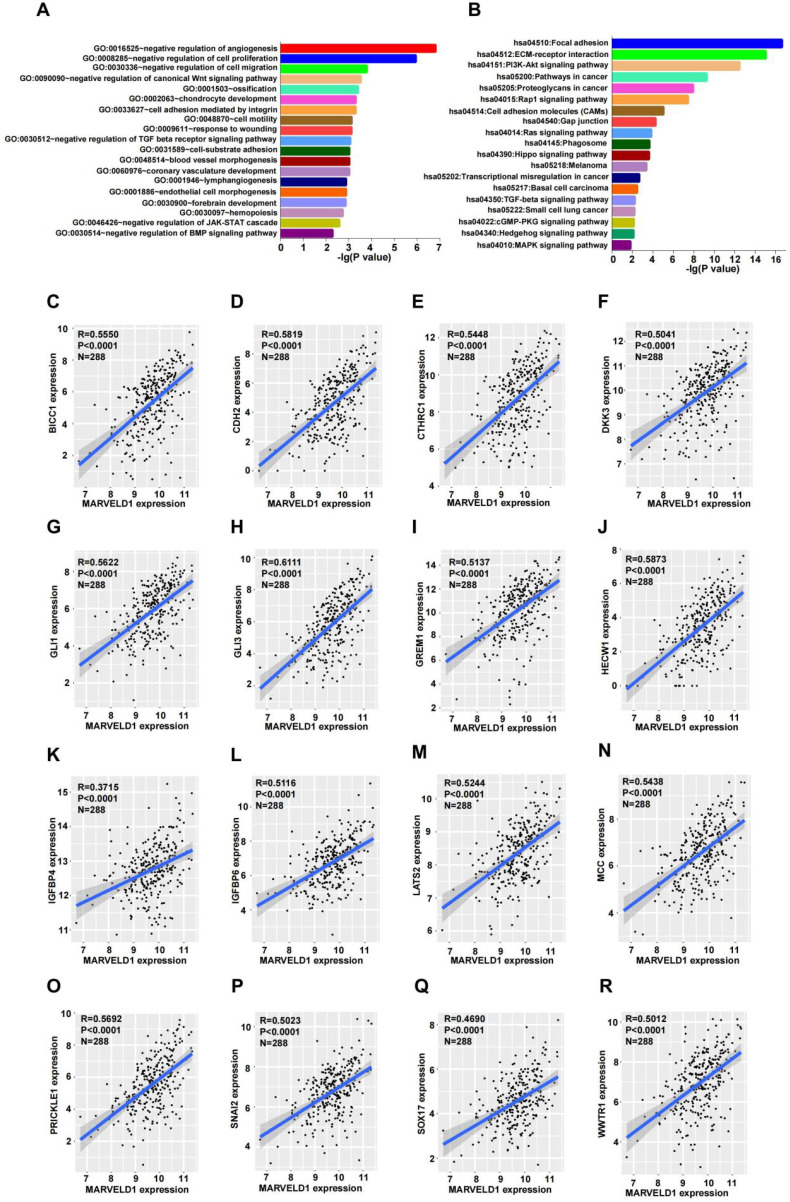
** MARVEL domain-containing 1 (MARVELD1) is associated with biological processes in colon cancer. (A-B)** Gene Ontology (GO) function and Kyoto Encyclopedia of Genes and Genomes (KEGG) pathway enrichment analysis of MARVELD1 in colon cancer. **(C-R)** MARVELD1 expression correlates with BICC1, CDH2, CTHRC1, DKK3, GLI1, GLI3, GREM1, HECW1, IGFBP4, IGFBP6, LATS2, MCC, PRICKLE1, SNAI2, SOX17, WWTR1 from the Wnt signaling pathway. All statistical tests were two-tailed, and P < 0.05 indicated statistical significance.

To explore the possible functions of MARVELD1, guilt-by-association analyses were performed using the data from TCGA. Patients with low expression of MARVELD1 had poor prognosis than those with high expression levels in our colon cancer cohort. Therefore, we analyzed the data from TCGA for COAD for MARVELD1 related genes through Pairwise Pearson correlation. GO and KEGG pathways were analyzed for the potential functions and pathways of MARVELD1 and correspond to enriched genes in COAD. Based on this analysis, the main functions of MARVELD1 were to negatively regulate angiogenesis, cell proliferation, cell migration, cell adhesion, and cell motility. Further, the major pathways regulated via MARVELD1 included the canonical Wnt, TGF-beta receptor, JAK-STAT cascade, BMP, Rap1, Ras, Hippo, MAPK, and PI3K-Akt signaling pathways (Figure 5A,B). We analyzed the correlation of MARVELD1 with key genes of essential pathways in COAD. The expression of MARVELD1 was correlated with genes related to the canonical Wnt signaling pathway (BICC1, CDH2, CTHRC1, DKK3, GLI1, GLI3, GREM1, HECW1, IGFBP4, IGFBP6, LATS2, MCC, PRICKLE1, SNAI2, SOX17, and WWTR1) (Figure 5C-R); angiogenesis (COL4A2, DCN, ECSCR, GPR4, and NPR1) (Supplementary Figure 2A-E); BMP signaling pathway (CHRD, FSTL3, FZD1, HTRA3, and SPG20) (Supplementary Figure 2F-J); cell migration (CLIC4, DLC1, DPYSL3, and IGFBP5) (Supplementary Figure 3A-D); cell proliferation (ADAMTS1, ADARB1, BCL6, CD33, CDH13, and COL18A1) (Supplementary Figure 3E-J); JAK-STAT pathway (BGN, FLRT2, LRRC15, PODN, and SOCS5) (Supplementary Figure 4A-E); and TGF-beta receptor pathway (ASPN, CHST11, ENG, and HTRA1) (Supplementary Figure 4F-I).

### MARVELD1 inhibits tumor growth *in vivo* and* in vitro* in colon cancer

**Figure 6:**
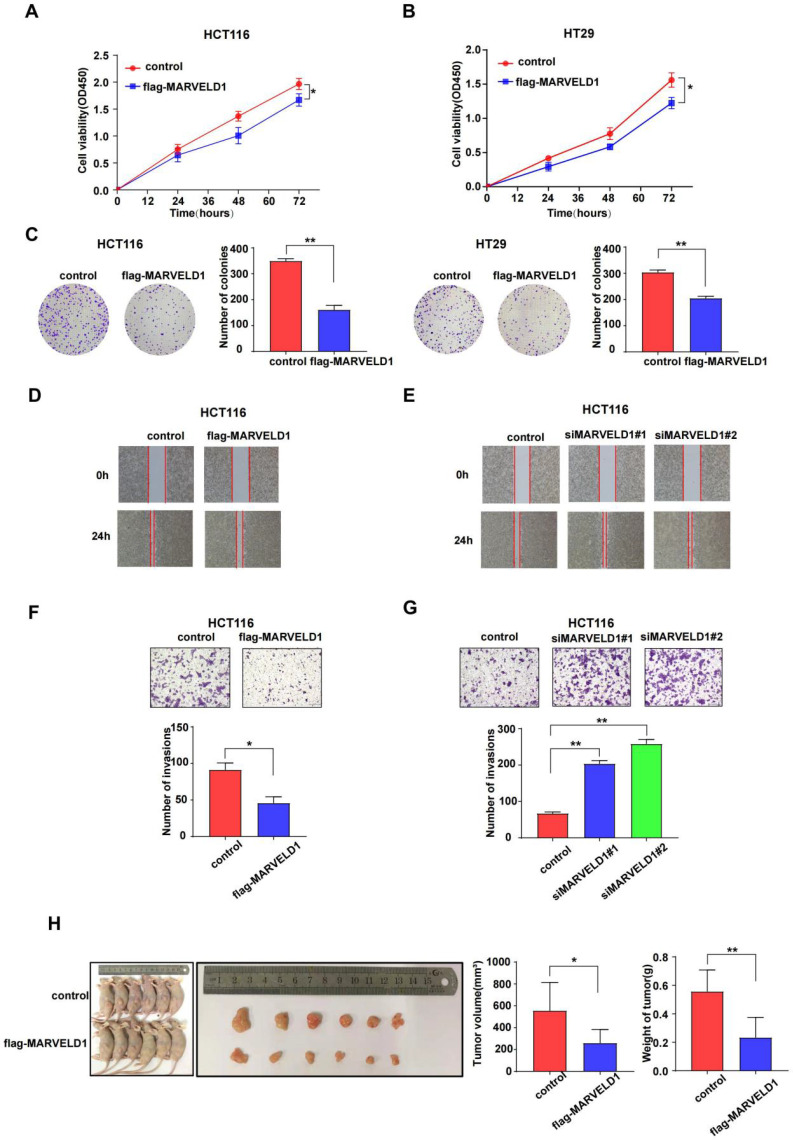
** MARVEL domain-containing 1 (MARVELD1) inhibites tumor cell proliferation, migration, and invasion, both *in vivo* and *in vitro*. (A-C)** The CCK-8 and the clonogenic assay indicate that overexpressing MARVELD1 inhibited the proliferation of HCT116 and HT29 cancer cells, respectively. **(D-G)** MARVELD1 inhibites migration and invasion in HCT116 cancer cell by the wound healing and the transwell assay. **(H)** The tumorigenesis and tumor tissue of each group of Balb/c mice (n = 6 per group) are shown. Tumor growth and tumor weight were measured for tumors in Balb/c mice induced by HCT116 cells transfected with an overexpression MARVELD1 lentivirus or control. Overexpression of MARVELD1 inhibited tumor growth compared with the controls. The median tumor weight in the MARVELD1 overexpression group was lower than that in the control group. *p< 0.05; **p < 0.01; ***p < 0.001; ****p < 0.0001; ns, no significance.

As the main functions and important pathways modulated via MARVELD1 were found in colon cancer when analyzed through GO and KEGG, colon cancer was selected for functional studies to elucidate the potential role of MARVELD1 in inhibiting tumorigenesis. MARVELD1 was stably overexpressed using purified flag-tagged lentivirus. Transient knockdown of MARVELD1 was achieved using two different siRNAs. In the HCT116 and HT29 cell lines, overexpression of MARVELD1 inhibited proliferation and clone formation as observed using the CCK-8 assay and clonogenic assay (all P < 0.05) (Figure 6A-C). In HCT116 and HT29 cell lines, overexpression of MARVELD1 decreased cell migration and invasion (all P < 0.05) (Figure 6D-G, Supplementary Figure 5). HCT116 cells, transfected with either control or overexpression MARVELD1 lentivirus, were examined to understand the role of MARVELD1 *in vitro*. Therefore, when BALB/c mice were injected with HCT116-controls or HCT116-flag-MARVELD1, the overexpression of MARVELD1 inhibited tumor growth compared with the controls. Moreover, the median tumor weight in the MARVELD1 overexpression group was lower than that in the control group (all P < 0.05) (Figure 6H). Therefore, MARVELD1 acts as a tumor suppressor and inhibits tumor growth in colon cancer.

### MARVELD1 suppressed activity of the Wnt/β-catenin pathway in colon cancer cells

**Figure 7:**
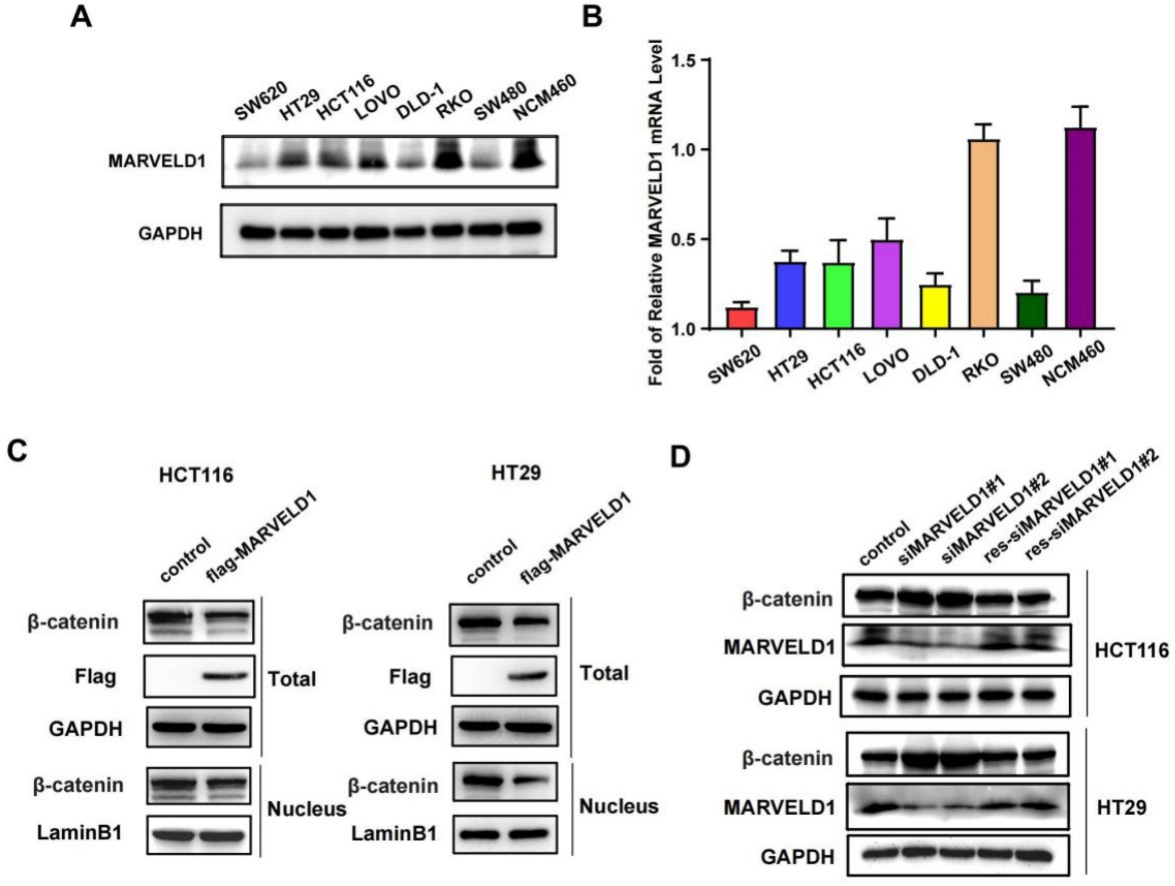
** The expression of MARVEL domain-containing 1 (MARVELD1) is downregulated in colon cancer cell lines in comparison to that in normal colon cell and regulates the Wnt/β-catenin pathway in HCT116 and HT29 cells. (A-B)** The expression of MARVELD1 is downregulated in SW620, HT29, HCT116, LoVo, DLD-1, RKO, and SW480 colon cancer cell lines in comparison to that in normal colon cells, i.e., NCM460, as observed by western blotting and qRT-PCR. **(C)** Overexpression of MARVELD1 inhibited β-catenin expression in HCT116 and HT29 cancer cells via western blot in the nucleus and in the total protein. **(D)** Knockdown of MARVELD1 increased β-catenin expression and then overexpressed, causing the expression of β-catenin to decrease in HCT116 and HT29 cancer cells via western blot.

The expression of *MARVELD1* was downregulated in seven colon cancer cell lines (SW620, HT29, HCT116, LoVo, DLD-1, RKO, and SW480) compared with normal colon cells, NCM460, at both mRNA and protein levels (Figure 7A-B). The activation of Wnt signaling caused the accumulation of β-catenin in the cytoplasm that was eventually translocated to the nucleus, which promotes the occurrence and progression of tumors (15, 16). We isolated the cytoplasm and nucleus from colon cancer cells and found that overexpression of MARVELD1 inhibited the expression of β-catenin in the nucleus and in the total protein (Figure 7C). The expression of MARVELD1 was knockdowned and then rescued, causing the expression of β-catenin to decrease at the protein levels (all P < 0.05) (Figure 7D).

### Function of MARVELD1 in colon cancer cells was mediated by the Wnt/β-catenin pathway

**Figure 8:**
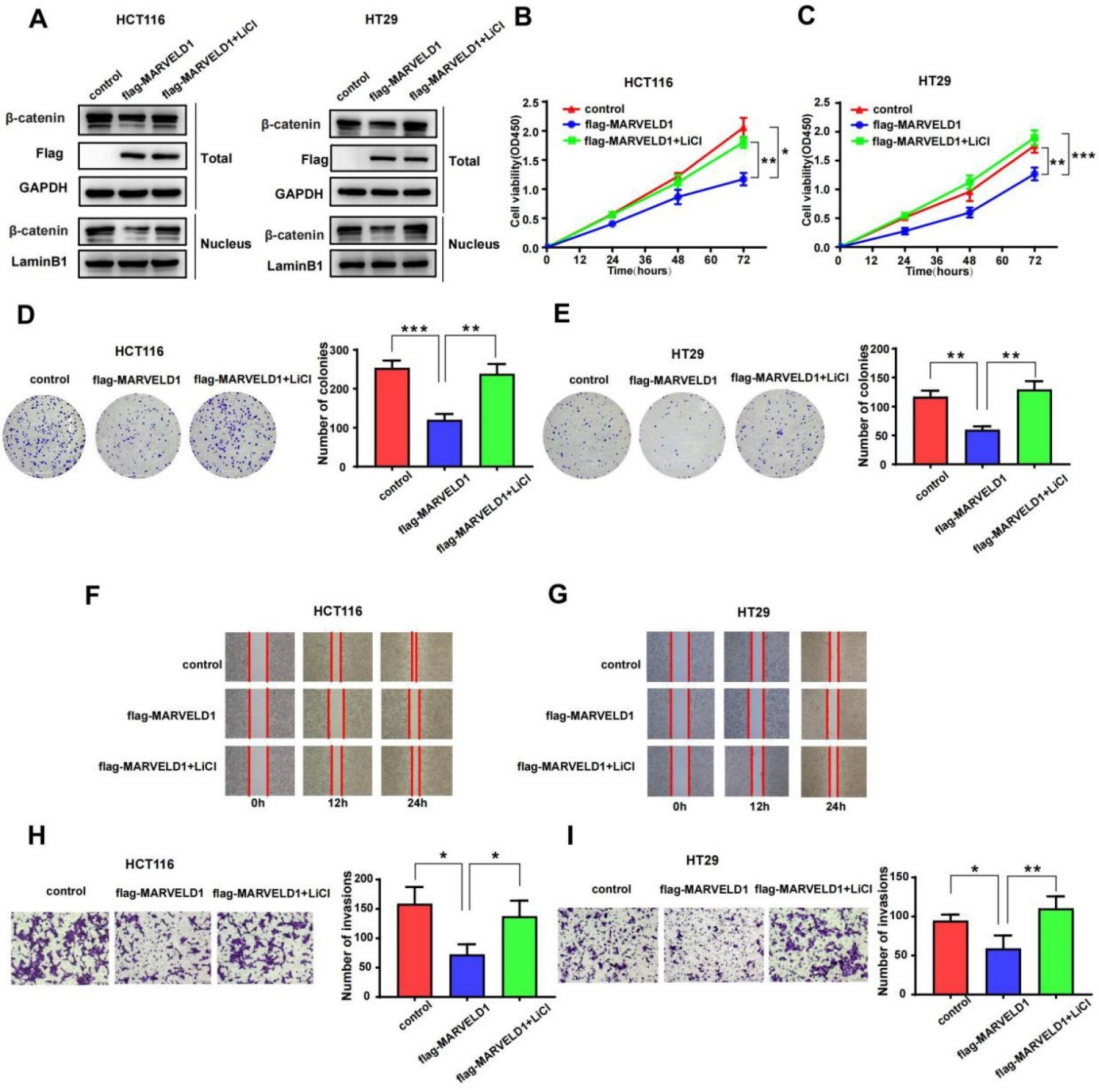
** Rescue experiments of MARVELD1. (A)** HCT116 and HT29 cells with MARVELD1 overexpression or control transfection were first incubated with or without 20 mmol/L LiCl and then the inhibition of β-catenin expression was partly reversed by western blotting in the nucleus and in the total protein. **(B-E)** HCT116 and HT29 cells with MARVELD1 overexpression or control transfection were first incubated with or without 20 mmol/L LiCl and then the inhibition of proliferation was partly reversed by the CCK-8 and clonogenic assays. **(F-I)** HCT116 and HT29 cells with MARVELD1 overexpression or control transfection were first incubated with or without 20 mmol/L LiCl and then the inhibition of migration and invasion were partly reversed by the wound healing and transwell assays. *p< 0.05; **p < 0.01; ***p < 0.001; ****p < 0.0001; ns, no significance.

To examine whether the function of MARVELD1 in colon cancer was mediated by the Wnt/β-catenin pathway, we used LiCl to activate the Wnt/β-catenin pathway in a rescue experiment 17. The role of MARVELD1 overexpression on the inhibition of β-catenin expression was partly reversed by LiCl in the total protein and the nucleus (Figure 8A). CCK-8 assay and clone formation showed that the inhibition of proliferation by MARVELD1 overexpression was partly reversed by LiCl in the HCT116 and HT29 cell lines (Figure 8B-E). The wound healing and the transwell assays showed that the inhibition of migration and invasion by MARVELD1 overexpression were partly reversed by LiCl (Figure 8F-I). All these results suggested that the role of MARVELD1 in colon cancer progression is mediated by the Wnt/β-catenin pathway.

## Discussion

The evolutionarily conserved MARVEL domain-containing proteins are downregulated in various types of cancer, and they exert tumor-suppressive functions 6-9. These proteins also play a role in several biological processes, including cell cycle progression, chemotactic activity, and clathrin-mediated endocytosis 18-20. MARVELD1, a novel member of this family, is widely expressed in normal human tissues but downregulated through promoter methylation in many primary tumors, including breast, cervical, prostate, hepatocellular, esophageal, gastric, and lung carcinomas or cancer cell lines 5, 21. In this study, first, we clarified the relationship between MARVELD1 expression and prognosis in pan-cancer from TCGA. Second, we analyzed the regulation upstream through epigenetic modification, and the transcription factors of MARVELD1 from pan-cancer data obtained from TCGA. Third, we elucidated the downstream regulatory functions of MARVELD1, the correlation between MARVELD1 and key genes of important pathways through GO and KEGG analysis in colon cancer. Fourth, we revealed that MARVELD1 regulated the occurrence and progression of tumors by inhibiting WNT/β-catenin signaling in colon cancer.

In this study, we showed that the expression of MARVELD1 was downregulated in pan-cancer, which is consistent with previous reports. The expression of *MARVELD1* was lower in colon cancer tissues than in their non-cancer tissue counterparts. MARVELD1 was mainly located in the nucleus in colon cancer, which was consistent with previous reports 5. TCGA data was used to validate the association of low expression of MARVELD1 with poor DSS and PFI in pan-cancer. Different types of cancers were also analyzed in our cohort. The expression of MARVELD1 was negatively correlated with both OS and PFS in COAD, and negatively correlated with OS in PRAD. Patients with non-small cell lung cancer (NSCLC) and liver cancer who had a poor prognosis exhibited low expression of MARVELD1 compared with those patients with high expression levels 11, 22. These results suggest that MARVELD1 has tumor suppressor activity.

The hypermethylation and demethylation of promoters of cancer-related genes are usually related to cell proliferation. Therefore, hypermethylation of tumor suppressor genes and demethylation of oncogenes contribute toward tumorigenesis 2, 3, 23. DNA methylation is reduced in the MARVELD1 promoter locus in multiple tumors and cell lines 4. MARVELD1 is synergistically inactivated through both DNA hypermethylation and histone deacetylation in lung cancer cells 18. Therefore, it is pertinent to study the epigenetic modifications that regulate MARVELD1 expression using pan-cancer data. Analyzing TCGA and 450K array databases, we predicted 13 methylation sites in the MARVELD1 promoter locus, which were hypermethylated in tissues from the pan-cancer data compared with those in non-cancer tissues. In most types of cancer, every methylation site in the MARVELD1 promoter locus was significantly hypermethylated than in their non-cancer counterparts. Moreover, our predicted methylation sites in pan-cancer were partially consistent with the previously verified methylation sites in lung cancer 21. We will verify 13 methylation sites of the MARVELD1 promoter locus in future colon cancer studies. In addition to DNA methylation, we also found that H3K27ac and H3K4me3 were enriched in the MARVELD1 promoter locus in ten cancer cell lines, including U87, MCF-7, LoVo, LNCaP, IMR90, HCT116, HCC1954, H128, Caco-2, and A549. Both H3K27Ac and H3K4me3 are generally correlated with gene activation. However, previous research reported that BAMBI, a tumor suppressor, was modified by H3K27ac 24. ZEB1 has tumor suppressor activity and is down-regulated in tumors, while H3K4me3 is also enriched in the promoter region of ZEB1 25, 26. The mechanism of gene transcription and expression is complex and affected by many factors, including DNA methylation and histone modification. Therefore, we hypothesized that MARVELD1, a tumor suppressor, is biphasically regulated via DNA methylation and histone modification, resulting in its reduced expression in cancer tissues. The lower levels of MARVELD1 expression were related to the comprehensive regulation of epigenetic factors, such as DNA methylation and histone acetylation. Transcription factors promote or inhibit gene expression by interacting with cis-acting factors. Transcription factors mainly interact with the transcription regulatory region, including the transcription activation and transcription repression domains. The transcription repression domain inhibits transcription in tumors. Therefore, in addition to studying methylation and histone modification of the MARVELD1 promoter region, we used the JASPAR software to predict upstream transcription factors. We screened five transcription factors, including FOSL1, JUN, NFATC1, NFAT3, and TCF7L2, which also related to the Wnt signaling pathway as shown by GO and KEGG analyses. Visualizing the ChIP‐seq data validated these five transcription factors in different cell lines using the ENCODE database. In the HCT116 cell lines, we predicted two transcription factors, FOSL1 and TCF7L2, bound to the MARVELD1 promoter locus. Previous studies reported that FOSL1 was downregulated in cervical carcinoma, and it inhibited the tumorigenicity of cervical carcinoma 27, 28. Moreover, loss of the nuclear Wnt pathway effector TCF7L2 promoted migration and invasion of human colorectal cancer cells 29. Previous reports indicated that both FOSL1 and TCF7L2 had tumor suppressor activity. In colon cancer, transcription factors regulate MARVELD1 expression, and MARVELD1 in turn decreases the cellular level of β-catenin. It means that MARVELD1 helps to construct a negative feedback loop for the Wnt/β-catenin pathway.

**Figure 9:**
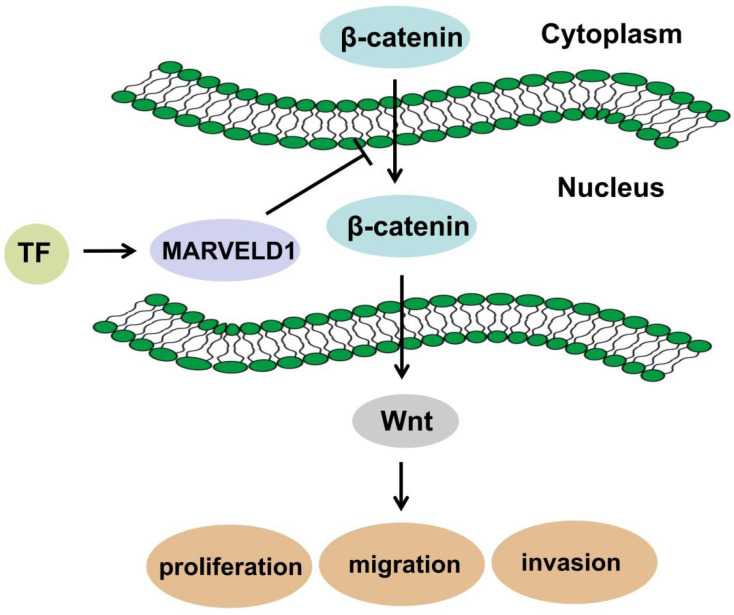
Schematic diagram of mechanism in which MARVELD1 inhibited Wnt/β-catenin pathway.

MARVELD1 is a microtubule-associated protein that can significantly inhibit cell proliferation, promote G1 phase cell-arrest, and reduce cell migration in mice 30. Moreover, MARVELD1 plays a role in the pre-mRNA processing of integrin β1, thereby regulating cell adhesion and cell movement, a process paralleled to nonsense-mediated mRNA decay (NMD) and associated with the potential NMD factor Importin b1 31-33. Reducing MARVELD1 levels in lung cancer tissues also reduces the efficiency of NMD by diminishing the link between the UPF1/SMG1 components of the NMD complex and the mRNA containing a premature stop codon (PTC-mRNA) 18, 34. These reports suggest that the function of MARVELD1 could be related to the NMD regulatory signaling pathway. Low expression of MARVELD1 was significantly associated with poor prognosis of colon cancer. Therefore, we analyzed the potential biological functions and pathways of MARVELD1 in COAD through the GO and KEGG pathway analyses. The results indicated that MARVELD1 also influenced angiogenesis, cell-substrate adhesion, blood vessel morphogenesis, lymphangiogenesis, and endothelial cell morphogenesis. Furthermore, MARVELD1 negatively regulates many tumor-related signaling pathways in COAD, including canonical Wnt, TGF-beta receptor, JAK-STAT cascade, BMP, Rap1, Ras, Hippo, MAPK, and PI3K-Akt signaling pathways. In this study, MARVELD1 inhibited cell proliferation, migration, and invasion *in vitro* and inhibited proliferation *in vivo* in colon cancer cells. These results indicate that MARVELD1 is a tumor suppressor in colon cancer. As the upstream transcriptional regulation and downstream functional enrichment of MARVELD1 indicated the Wnt signaling pathway, we analyzed this potential relationship through molecular biology experiments. Studies have shown that the Wnt pathway in cells is mainly composed of the following proteins: β-catenin, adenoma-tous poly posis coli protein (APC), glycogen synthase kinase-3β (GSK-3β) and Axin or conductin. β-catenin is a positive regulator of Wnt pathway, while APC protein, GSK-3β and Axin are negative regulators 35, 36. The activation of this pathway enables β-catenin to accumulate in the cytoplasm and eventually translocate to the nucleus, where it serves as a co-activator of the TCF/LEF family of transcription factors, which promotes tumor formation and progression 13, 14. Therefore, we tested whether MARVELD1 inhibited β-catenin translocation to the nucleus. We observed that MARVELD1 negatively regulated β-catenin expression at the protein level. In addition, we observed that overexpression of MARVELD1 downregulated β-catenin expression in the nucleus and in the total protein. To examine whether the function of MARVELD1 in colon cancer was mediated by the Wnt/β-catenin pathway, we used LiCl to activate the Wnt/β-catenin pathway in a rescue experiment 17. The role of MARVELD1 overexpression on the inhibition of β-catenin expression was partly reversed by LiCl in the total protein and the nucleus in the HCT116 and HT29 cells. LiCl significantly enhanced proliferation, migration, and invasion of colon cancer cells overexpressing MARVELD1. The knockout of MARVELD1 upregulates p-ERK1/2 and cyclinD1 and downregulates p16 and p53 10. CyclinD1 is a classic target gene downstream of the Wnt/β-catenin signaling pathway 37-39. Therefore, we infer that the knockout of MARVELD1 might increase CyclinD1 levels through the Wnt/β-catenin signaling pathway. High MARVELD1 expression reduces integrin b1 levels and inhibits cell migration and EMT in NSCLC cells 11. The Wnt signaling pathway can induce EMT conversion by inhibiting the degradation of β-catenin 40-42. In colon cancer, transcription factors regulate MARVELD1 expression, and MARVELD1 subsequently decreases the cellular level of β-catenin. This shows that MARVELD1 helps to construct a negative feedback loop involving the Wnt/β-catenin pathway (Figure 9). Therefore, we conclude that MARVELD1 regulates the occurrence and progression of tumors through the Wnt/β-catenin signaling pathway.

MARVELD1 is also associated with sensitivity to chemotherapy. The overexpression of MARVELD1 enhanced the chemotherapeutic sensitivity of liver cancer cells to epirubicin and 10-hydroxycamptothecin 10. Low expression of MARVELD1 was also related to ovarian cancer platinum- and taxane-based chemotherapy resistance 43. In addition, MARVELD1 interacts with catalase (CAT) to regulate the proliferation of epithelial tumor cells of the reproductive system and the sensitivity to chemotherapy drugs by reducing excessive ROS 44.

## Conclusion

Overall, MARVELD1—a tumor suppressor that is epigenetically modified—inhibits the occurrence and progression of tumors through the Wnt/β-catenin signaling pathway.

## Supplementary Material

Supplementary figures and tables.

## Figures and Tables

**Table 1:** Methylation of MARVELD1 locus between cancer and normal tissues in Pan-cancer types

Cancer type	cg site	Number of tumor and normal tissues	P value
Pan-cancer	cg18744118	Num(T)=8916;Num(N)=723	ns
	cg23733123	Num(T)=8916;Num(N)=723	***
	cg25355803	Num(T)=8916;Num(N)=723	****
	cg27165884	Num(T)=8916;Num(N)=723	****
	cg06679087	Num(T)=8916;Num(N)=723	***
	cg06616710	Num(T)=8916;Num(N)=723	****
	cg00844376	Num(T)=8915;Num(N)=723	****
	cg24576735	Num(T)=8915;Num(N)=723	****
	cg26181880	Num(T)=8916;Num(N)=723	***
	cg16381596	Num(T)=8916;Num(N)=722	****
	cg15681358	Num(T)=8916;Num(N)=723	***
	cg24500959	Num(T)=8916;Num(N)=723	*
	cg19201009	Num(T)=8914;Num(N)=723	**
	cg22756156	Num(T)=8916;Num(N)=723	****

Notes: * p<0.05, ** p<0.01, *** p<0.001, **** p<0.0001, ns: no significance.

**Table 2:** Methylation of MARVELD1 locus between cancer and normal tissues in different cancer types

cg site	Cancer type	Number of tumor and normal tissues	P value
cg23733123	BLCA	Num(T)=413;Num(N)=21	ns
	BRCA	Num(T)=790;Num(N)=98	****
	CESC	Num(T)=309;Num(N)=3	ns
	COAD	Num(T)=299;Num(N)=38	ns
	LUAD	Num(T)=460;Num(N)=32	ns
	PRAD	Num(T)=499;Num(N)=50	****
	UCEC	Num(T)=432;Num(N)=46	****
	KIRP	Num(T)=276;Num(N)=45	****
cg25355803	BLCA	Num(T)=413;Num(N)=21	ns
	BRCA	Num(T)=790;Num(N)=98	****
	CESC	Num(T)=309;Num(N)=3	ns
	COAD	Num(T)=299;Num(N)=38	**
	LUAD	Num(T)=460;Num(N)=32	ns
	PRAD	Num(T)=499;Num(N)=50	****
	UCEC	Num(T)=432;Num(N)=46	****
	KIRP	Num(T)=276;Num(N)=45	****
	THCA	Num(T)=515;Num(N)=56	****
	KIRC	Num(T)=320;Num(N)=160	****
cg27165884	BRCA	Num(T)=790;Num(N)=98	****
	CESC	Num(T)=309;Num(N)=3	ns
	COAD	Num(T)=299;Num(N)=38	*
	LUAD	Num(T)=460;Num(N)=32	**
	PRAD	Num(T)=499;Num(N)=50	****
	UCEC	Num(T)=432;Num(N)=46	*
	KIRP	Num(T)=276;Num(N)=45	**
	LIHC	Num(T)=379;Num(N)=50	****
cg06679087	BRCA	Num(T)=790;Num(N)=98	****
	LUAD	Num(T)=460;Num(N)=32	**
	PRAD	Num(T)=499;Num(N)=50	****
	UCEC	Num(T)=432;Num(N)=46	***
	LIHC	Num(T)=379;Num(N)=50	***
cg06616710	BRCA	Num(T)=790;Num(N)=98	****
	CESC	Num(T)=309;Num(N)=3	ns
	COAD	Num(T)=299;Num(N)=38	ns
	LUAD	Num(T)=460;Num(N)=32	***
	PRAD	Num(T)=499;Num(N)=50	****
	UCEC	Num(T)=432;Num(N)=46	ns
	KIRP	Num(T)=276;Num(N)=45	**
	LIHC	Num(T)=379;Num(N)=50	****
	THCA	Num(T)=515;Num(N)=56	*
cg00844376	BRCA	Num(T)=790;Num(N)=98	**
	LUSC	Num(T)=372;Num(N)=43	****
	HNSC	Num(T)=530;Num(N)=50	***
	KIRC	Num(T)=320;Num(N)=160	**
cg16381596	BRCA	Num(T)=790;Num(N)=98	****
	CESC	Num(T)=309;Num(N)=3	ns
	COAD	Num(T)=299;Num(N)=38	ns
	LUSC	Num(T)=372;Num(N)=43	****
	PAAD	Num(T)=185;Num(N)=10	****
	HNSC	Num(T)=530;Num(N)=50	**
	KIRC	Num(T)=320;Num(N)=160	****
cg15681358	BRCA	Num(T)=790;Num(N)=98	****
	CESC	Num(T)=309;Num(N)=3	ns
	COAD	Num(T)=299;Num(N)=38	ns
	PRAD	Num(T)=499;Num(N)=50	****
	UCEC	Num(T)=432;Num(N)=46	**
	LIHC	Num(T)=379;Num(N)=50	****
cg24500959	BRCA	Num(T)=790;Num(N)=98	****
	CESC	Num(T)=309;Num(N)=3	ns
	LUAD	Num(T)=460;Num(N)=32	****
	PRAD	Num(T)=499;Num(N)=50	****
	UCEC	Num(T)=432;Num(N)=46	****
	LIHC	Num(T)=379;Num(N)=50	***
cg19201009	BLCA	Num(T)=413;Num(N)=21	***
	BRCA	Num(T)=790;Num(N)=98	****
	CESC	Num(T)=309;Num(N)=3	*
	LUAD	Num(T)=460;Num(N)=32	*
	PRAD	Num(T)=499;Num(N)=50	****
	UCEC	Num(T)=432;Num(N)=46	****
	KIRP	Num(T)=276;Num(N)=45	****
	HNSC	Num(T)=530;Num(N)=50	**

Notes: * p<0.05, ** p<0.01, *** p<0.001, **** p<0.0001, ns: no significance.

## Abbreviations

DSS: Disease Specific Survival
PFI: Progression free interval
PFS: Progression Free Survival
OS: Overall survival
ACC: Adrenocortical carcinoma
BLCA: Bladder Urothelial Carcinoma
BRCA: Breast invasive carcinoma
CESC: Cervical squamous cell carcinoma and endocervical adenocarcinoma
CHOL: Cholangio carcinoma
COAD: Colon adenocarcinoma
DLBC: Lymphoid Neoplasm Diffuse Large B-cell Lymphoma
ESCA: Esophageal carcinoma
GBM: Glioblastoma multiforme
HNSC: Head and Neck squamous cell carcinoma
KICH: Kidney Chromophobe
KIRC: Kidney renal clear cell carcinoma
KIRP: Kidney renal papillary cell carcinoma
LAML: Acute Myeloid Leukemia
LGG: Brain Lower Grade Glioma
LIHC: Liver hepatocellular carcinoma
LUAD: Lung adenocarcinoma
LUSC: Lung squamous cell carcinoma
MESO: Mesothelioma
OV: Ovarian serous cystadenocarcinoma
PAAD: Pancreatic adenocarcinoma
PCPG: Pheochromocytoma and Paraganglioma
PRAD: Prostate adenocarcinoma
READ: Rectum adenocarcinoma
SARC: Sarcoma
SKCM: Skin Cutaneous Melanoma
STAD: Stomach adenocarcinoma
TGCT: Testicular Germ Cell Tumors
THCA: Thyroid carcinoma
THYM: Thymoma
UCEC: Uterine Corpus Endometrial Carcinoma
UCS: Uterine Carcinosarcoma
UVM: Uveal Melanoma
