# Supplementary material for: Epigenetic modifications inhibit the expression of MARVELD1 and in turn tumorigenesis by regulating the Wnt/β-catenin pathway in pan-cancer
Source: J Cancer. 2022 Jan 1;13(1):225–42. doi: 10.7150/jca.63608 (PMC8692698; doi:10.7150/jca.63608)
Supplement: Supplementary file 1 — Supplementary figures and tables. [file jcav13p0225s1.pdf]

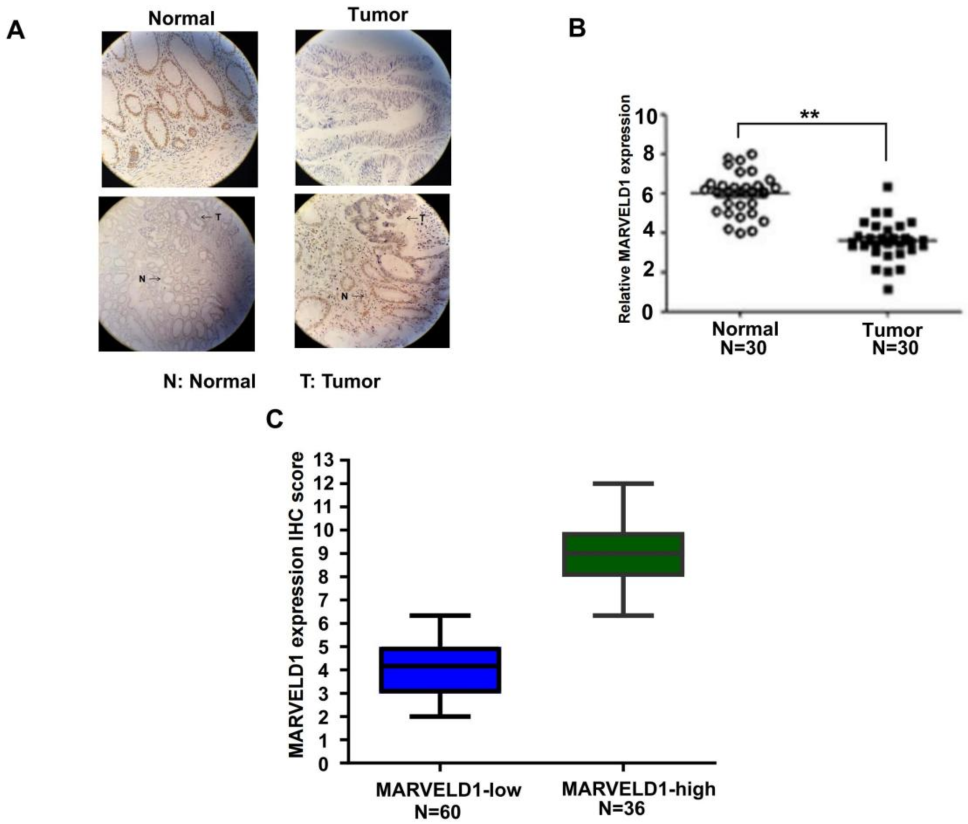


Supplementary FIGURE 1: MARVEL domain-containing 1 (MARVELD1) expression is downregulated in colon cancer. (A) The expression of MARVELD1 protein in colon cancer tissues and adjacent tissues by immunohistochemistry. (B) MARVELD1 expression is lower in colon cancer tissues than normal tissues in our cohort via qRT-PCR. (C) Colon cancer tissues were divided into MARVELD1 high expression group (n = 36) and low expression group (n = 60) according to the expression difference of MARVELD1 in colon cancer by immunohistochemistry. *p< 0.05; **p < 0.01; ***p < 0.001; ****p < 0.0001; ns, no significance.

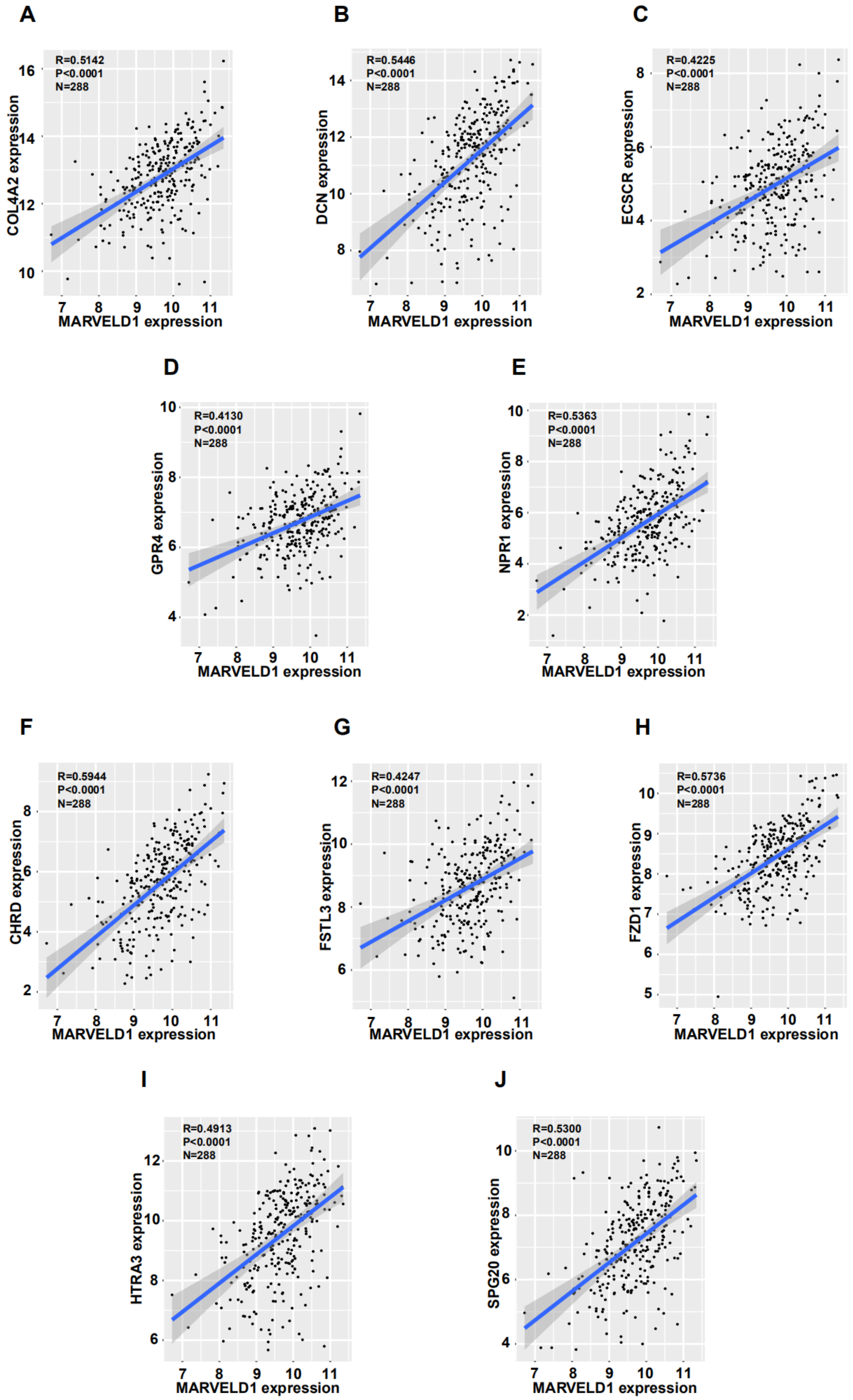
A
R=0.5142
P<0.0001
N=288
COL4A2 expression
MARVELD1 expression
B
R=0.5446
P<0.0001
N=288
DCN expression
MARVELD1 expression
C
R=0.4225
P<0.0001
N=288
ECSCR expression
MARVELD1 expression
D
R=0.4130
P<0.0001
N=288
GPR4 expression
MARVELD1 expression
E
R=0.5363
P<0.0001
N=288
NPR1 expression
MARVELD1 expression
F
R=0.5944
P<0.0001
N=288
CHRD expression
MARVELD1 expression
G
R=0.4247
P<0.0001
N=288
FSTL3 expression
MARVELD1 expression
H
R=0.5736
P<0.0001
N=288
FZD1 expression
MARVELD1 expression
I
R=0.4913
P<0.0001
N=288
HTRA3 expression
MARVELD1 expression
J
R=0.5300
P<0.0001
N=288
SPG20 expression
MARVELD1 expression

Supplementary FIGURE 2: MARVEL domain-containing 1 (MARVELD1) expression correlates with angiogenesis and the BMP signaling pathway. (A–E) MARVELD1 expression correlates with COL4A2, DCN, ECSCR, GPR4, and NPR1, which are related to signaling that mediate angiogenesis. (F–J) MARVELD1 expression correlates with CHRD, FSTL3, FZD1, HTRA3, and SPG20 from the BMP signaling pathway. All statistical tests were two-tailed, and $P < 0.05$ indicated statistical significance.

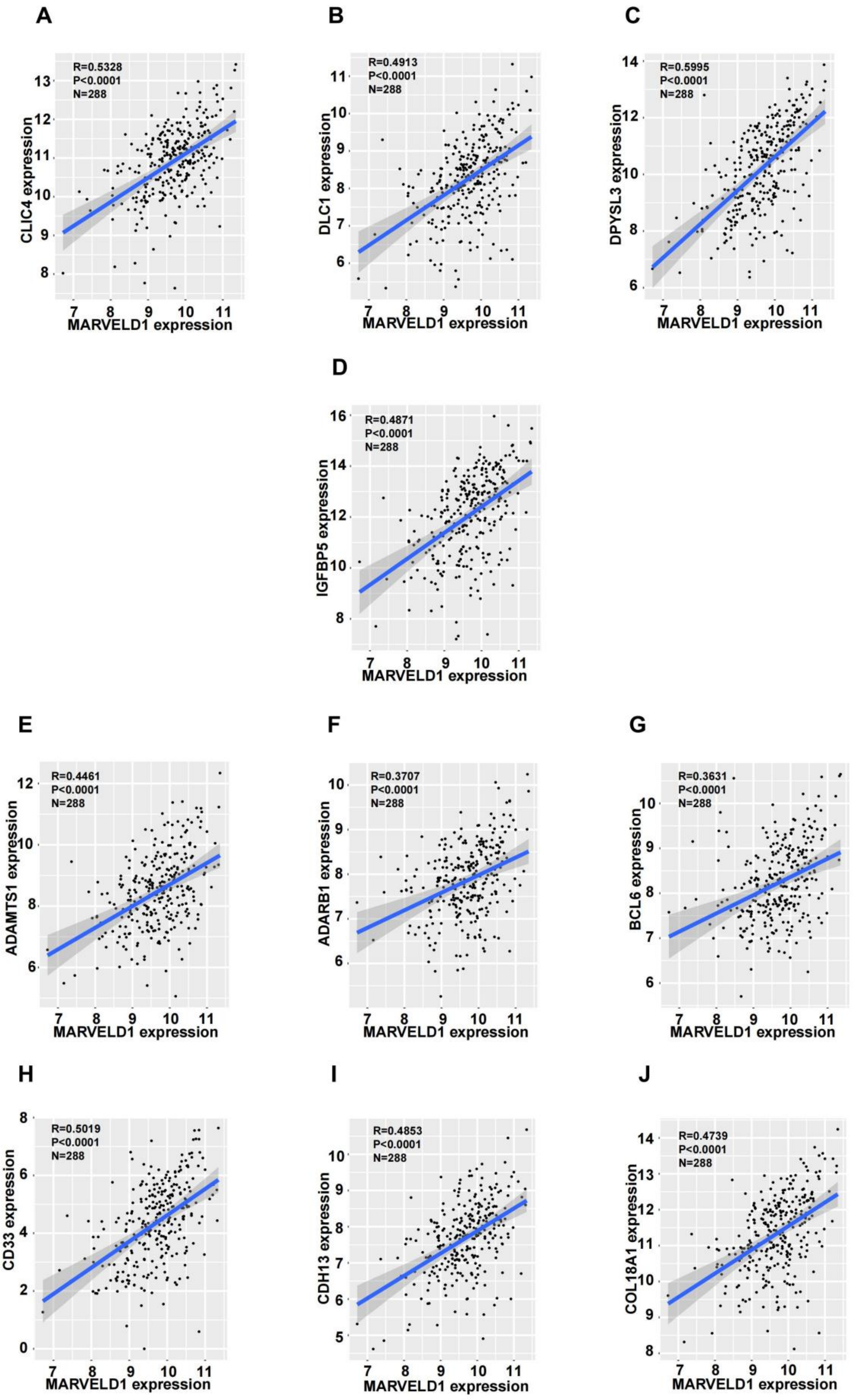


Supplementary FIGURE 3: MARVEL domain-containing 1 (MARVELD1) expression correlates with cell migration and cell proliferation. (A–D) MARVELD1

expression correlates with CLIC4, DLC1, DPYSL3, and IGFBP5, which are related to control cell migration. (E–J) MARVELD1 expression correlates with ADAMTS1, ADARB1, BCL6, CD33, CDH13, and COL18A1, which are related to mediate cell proliferation. All statistical tests were two-tailed, and $P < 0.05$ indicated statistical significance.

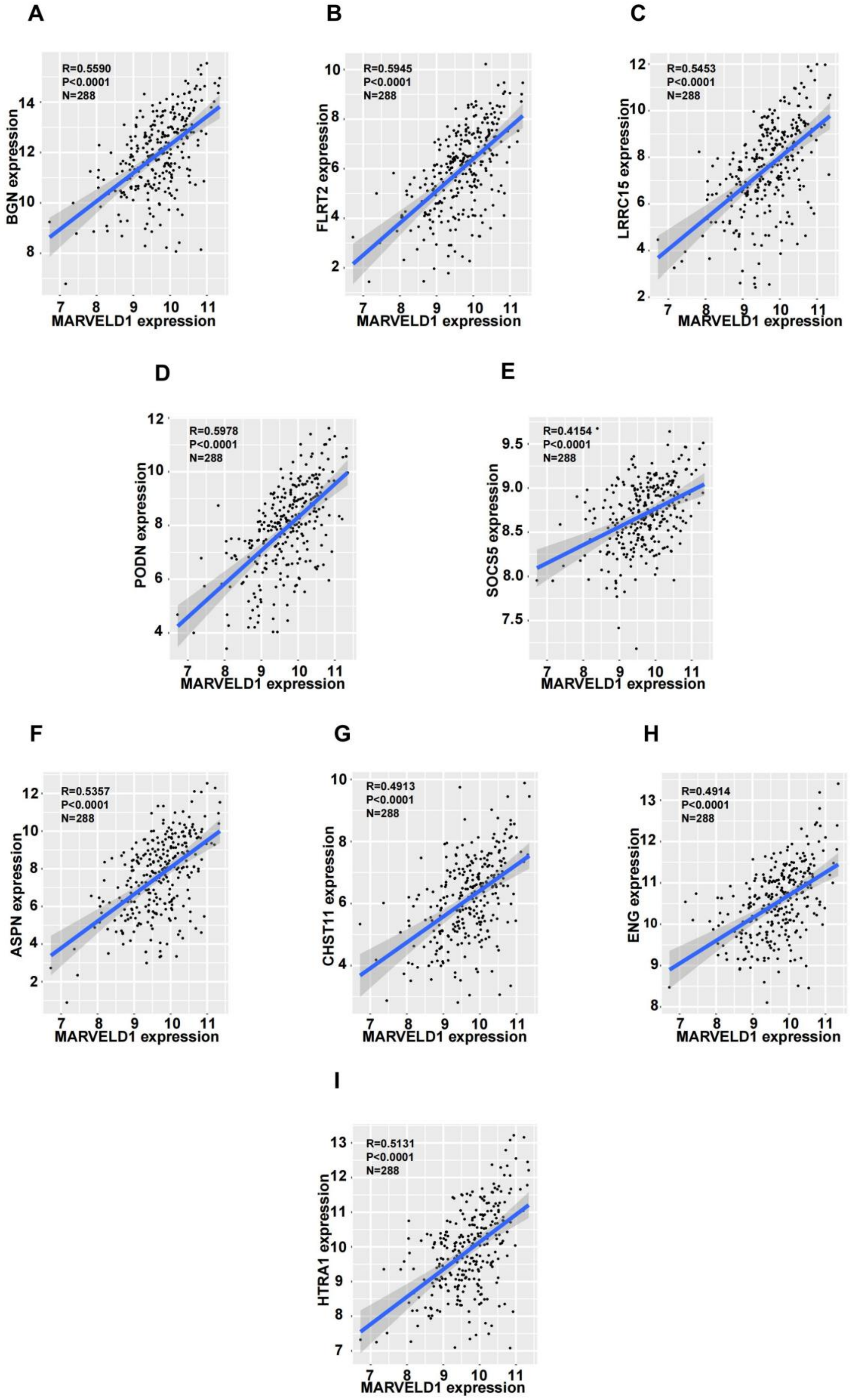
A
R=0.5590
P<0.0001
N=288
BGN expression
MARVELD1 expression
B
R=0.5945
P<0.0001
N=288
FLRT2 expression
MARVELD1 expression
C
R=0.5453
P<0.0001
N=288
LRRC15 expression
MARVELD1 expression
D
R=0.5978
P<0.0001
N=288
PODN expression
MARVELD1 expression
E
R=0.4154
P<0.0001
N=288
SOCS5 expression
MARVELD1 expression
F
R=0.5357
P<0.0001
N=288
ASPN expression
MARVELD1 expression
G
R=0.4913
P<0.0001
N=288
CHST11 expression
MARVELD1 expression
H
R=0.4914
P<0.0001
N=288
ENG expression
MARVELD1 expression
I
R=0.5131
P<0.0001
N=288
HTRA1 expression
MARVELD1 expression

Supplementary FIGURE 4: MARVEL domain-containing 1 (MARVELD1) expression correlates with the JAK-STAT cascade and the TGF-β receptor signaling pathway. (A–E) MARVELD1 expression correlates with BGN, FLRT2, LRRC15, PODN, and SOCS5 from the JAK-STAT cascade signaling pathway. (F–I) MARVELD1 expression correlates with ASPN, CHST11, ENG, and HTRA1 from the TGF-β receptor signaling pathway. All statistical tests were two-tailed, and P < 0.05 indicated statistical significance.

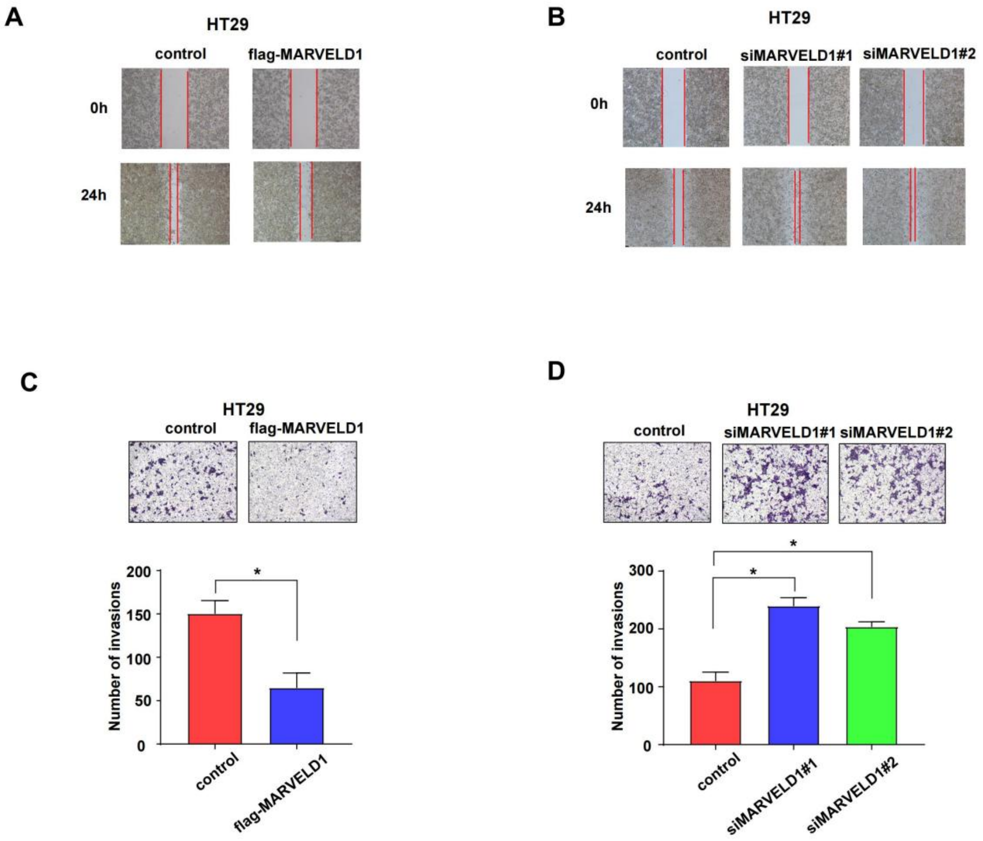


Supplementary FIGURE 5: MARVEL domain-containing 1 (MARVELD1) inhibites migration and invasion in HT29 cancer cell. *p< 0.05; **p < 0.01; ***p < 0.001; ****p < 0.0001; ns, no significance.

Supplementary Table 1 Expression of MARVELD1 between cancer and normal tissues in different cancer types

| Cancer type | Number of tumor and normal | P value |
|---|---|---|
| BLCA | Num(T)=407;Num(N)=19 | ** |
| BRCA | Num(T)=1104;Num(N)=114 | **** |
| CESC | Num(T)=305;Num(N)=3 | ** |
| COAD | Num(T)=288;Num(N)=41 | *** |
| KICH | Num(T)=66;Num(N)=25 | **** |
| LIHC | Num(T)=373;Num(N)=50 | ** |
| LUAD | Num(T)=517;Num(N)=59 | ** |
| LUSC | Num(T)=502;Num(N)=51 | ns |
| PAAD | Num(T)=179;Num(N)=4 | ns |
| PRAD | Num(T)=498;Num(N)=52 | **** |
| THYM | Num(T)=120;Num(N)=2 | ns |
| UCEC | Num(T)=177;Num(N)=24 | **** |
| Pan-cancer | Num(T)=9649;Num(N)=885 | *** |

Notes： * p<0.05, ** p<0.01,*** p<0.001,**** p<0.0001, ns: no significance

Supplementary Table 2 Relationship between MARVELD1 and clinicopathological parameters in the colon cancer.

| Parameter | Total | MARVELD1 expression | | P value |
|---|---|---|---|---|
| | | Low | High | |
| All patients | 96 | 60 | 36 | |
| Gender | | | | |
| male | 59 | 37 | 22 | 0.957 |
| Female | 37 | 23 | 14 | |
| Age（years） | | | | |
| ＜60 | 57 | 37 | 20 | 0.555 |
| ≥60 | 39 | 23 | 16 | |
| Histological differentiation | | | | |
| Well/Moderately | 72 | 42 | 30 | 0.144 |
| Poorly | 24 | 18 | 6 | |
| Depth of invasion | | | | |
| T3 | 46 | 33 | 13 | 0.073 |
| T4 | 50 | 27 | 23 | |
| Lymph node metastasis | | | | |
| N0 | 31 | 20 | 11 | 0.256 |
| N1 | 29 | 21 | 8 | |

| | | | | |
|---|---|---|---|---|
| N2 | 36 | 19 | 17 | |
| Distant metastasis | | | | |
| M0 | 29 | 16 | 13 | 0.329 |
| M1 | 67 | 44 | 23 | |
| Pathologic stage | | | | |
| II | 15 | 7 | 8 | |
| III | 14 | 9 | 5 | 0.385 |
| IV | 67 | 44 | 23 | |

Supplementary Table 3 Relationship between MARVELD1 and clinicopathological parameters in the pancreatic cancer.

| Parameter | Total | MARVELD1 expression | | P value |
|---|---|---|---|---|
| | | Low | High | |
| All patients | 37 | 23 | 14 | |
| Gender | | | | |
| male | 26 | 17 | 9 | 0.534 |
| Female | 11 | 6 | 5 | |
| Age（years） | | | | |

| | | | | |
|---|---|---|---|---|
| ＜60 | 20 | 11 | 9 | 0.33 |
| ≥60 | 17 | 12 | 5 | |
| Histological differentiation | | | | |
| Well/Moderately | 19 | 10 | 9 | 0.219 |
| Poorly | 18 | 13 | 5 | |
| Depth of invasion | | | | |
| T1-T2 | 11 | 5 | 6 | 0.173 |
| T3-T4 | 26 | 18 | 8 | |
| Lymph node metastasis | | | | |
| N0 | 29 | 17 | 12 | 0.398 |
| N1 | 8 | 6 | 2 | |
| Distant metastasis | | | | |
| M0 | 33 | 20 | 13 | 0.575 |
| M1 | 4 | 3 | 1 | |
| Pathologic stage | | | | |
| I | 7 | 2 | 5 | 0.212 |
| II | 14 | 9 | 5 | |
| III | 12 | 9 | 3 | |
| IV | 4 | 3 | 1 | |
